# Severity Stratification Changes What Hallucination Rates Mean: An Item-Matched Audit of Five Large Language Models in Orthodontic Decision Support

**DOI:** 10.3390/diagnostics16193180

**Published:** 2026-09-30

**Authors:** Ferdi Allaf, Mustafa Ozcan

**Affiliations:** Department of Orthodontics, Faculty of Dentistry, Istanbul Health and Technology University, İmrahor Cad. No. 82, Sütlüce, Beyoğlu, 34275 Istanbul, Türkiye; ferdi.allaf@istun.edu.tr

**Keywords:** artificial intelligence, large language models, orthodontics, patient safety, clinical decision support, health information quality

## Abstract

**Background/Objectives:** Large language models (LLMs) are consulted for clinical decision support, and their reliability is judged by hallucination prevalence—a measure treating every unsupported element as equivalent, although a fabricated citation and a fabricated protocol differ in what a clinician acting on them would do. We tested whether prevalence tracks clinical risk. **Methods:** Five LLMs answered a 100-item orthodontic benchmark validated by three external orthodontists (content validity index 0.923). The reference standard was fixed by construction for the 45 items naming a non-existent entity, so any substantive elaboration is unsupported by design; citations were adjudicated against PubMed and CrossRef. Responses were coded with a seven-category taxonomy and assigned to clinical, operational, or epistemic severity tiers in a post hoc exploratory stratification, pre-specified rather than prospectively registered. Because all models answered the same items, comparisons used Cochran’s Q with pairwise McNemar tests and generalised estimating equations clustered on item. **Results:** Of 500 responses, 449 (89.8%) contained a hallucination but only 65 (13.0%; 95% CI 10.3–16.2) were clinically consequential: prevalence was roughly sevenfold greater than the rate of clinically consequential output as the authors defined it. Between-model differences were large for undifferentiated prevalence (73–100%; Q = 50.51, *p* < 0.001) and contracted at the clinical tier (10–16%; Q = 10.00, *p* = 0.040), where no pairwise contrast survived adjustment. Item-level clustering was far stronger for clinically consequential output than for undifferentiated prevalence (intra-class correlation 0.83 versus 0.07). **Conclusions:** Prevalence and clinically consequential error are not interchangeable and rank models differently. The stratification is exploratory and author-defined, and the benchmark stress-tests susceptibility to fabricated premises rather than surveying natural use.

## 1. Introduction

Large language models (LLMs) are now consulted for clinical information by practitioners, trainees, and patients, and proposals to embed them in decision-support workflows depend on some estimate of how reliable their output is. The estimate almost universally used is hallucination prevalence: the proportion of responses containing content unsupported by any source [1,2]. In medical and dental applications, the reported rates depend on how a fabricated reference is defined [3,4]. One analysis of ChatGPT-generated medical content found 47% of references fabricated outright, a further 46% authentic but inaccurate, and only 7% both authentic and accurate [5]. Another, examining 178 references, found that 38.8% carried a non-functional or misattributed DOI and that 15.7% could not be located at all [6]. Comparable behaviour has been described in systematic review support [7]. Orthodontics is no exception: comparative evaluations have found accuracy that varies by model and by topic [8,9,10,11], chatbot performance on orthodontic multiple-choice items has ranged from 72% to 85% with highly variable concordance against orthodontists [12], and a recent meta-analysis reported wide heterogeneity across studies [13].

These studies share a measurement convention, and it is the convention rather than any individual result that concerns us here. A response is classified as hallucinated if it contains any unsupported element, regardless of what that element is. Under this convention, a response citing a non-existent reference while giving otherwise sound advice is counted identically to one describing a treatment protocol that does not exist. The two differ in what a clinician acting on them would do: the first obstructs verification, the second can change a treatment decision.

The convention has a measurable consequence. Citation-related errors are by far the most frequent category, so aggregate prevalence is dominated by them, and any model ranking derived from aggregate prevalence is largely a ranking of citation behaviour. Whether such rankings track clinical safety has not, to our knowledge, been tested.

This gap has been identified from within the orthodontic literature. Comparing five chatbots against 458 dental practitioners, Martina and colleagues found that accuracy tracked the degree of consensus in the underlying evidence, with the lowest educational quality on contested topics [14]. They noted two constraints on their design: that inadequate content may be camouflaged by fluent language—hallucination in the sense used here—which they could not measure, and that no verifiable gold standard existed against which to score responses, leaving expert judgement as the only reference.

That second constraint is not incidental. In a scenario-based comparison of four patient-facing chatbots against an expert benchmark in orthodontics, the three orthodontists constituting the benchmark agreed only moderately among themselves (Fleiss κ = 0.491) [15]. Where the reference standard carries that degree of internal disagreement, a confidently stated error and a confidently stated truth are difficult to separate, and the resulting estimate of chatbot accuracy inherits the uncertainty of the panel rather than measuring the model.

A second issue concerns what counts as a fabricated citation. Automated verification against bibliographic databases [16,17] returns three outcomes: confirmed match, confirmed absence, and indeterminate. Treating indeterminate results as fabrications conflates a property of the verification pipeline with a property of the model.

We addressed both issues in a domain chosen for its tractable ground truth. Orthodontics permits construction of items whose correct answer is fixed by design—an appliance or protocol that does not exist cannot be correctly elaborated—which removes the dependence on expert judgement that constrains most comparative evaluations. The methodological gap we address is therefore specific: prevalence has been measured repeatedly, but no study has asked whether the quantity it measures is the quantity that matters clinically, or whether rankings built on it survive a severity distinction. We tested three propositions, labelled P1 to P3 so that they are not confused with the taxonomy categories H1 to H7 introduced in the Methods. P1: Undifferentiated prevalence and clinically consequential error differ substantially in magnitude. P2: Between-model differences established on prevalence do not carry over unchanged to the clinical tier. P3: Clinically consequential output is concentrated in responses to items carrying a false premise. The measurement question generalises beyond the specialty; the benchmark is what is specific to it.

## 2. Materials and Methods

### 2.1. Study Design

This was a cross-sectional comparative evaluation of LLM-generated orthodontic content. Reporting follows the CHART statement for chatbot health advice studies [18]. STROBE [19] was written for studies of human participants. We applied only those items that transfer to a benchmark whose units of observation are model responses: study design, setting and dates, eligibility and selection of units, outcome definition, sources of bias, study size, statistical methods, descriptive and outcome data, limitations, and generalisability. Items concerning participants, exposures, confounding, follow-up, and matching of individuals do not apply and are marked as such in the completed checklist (File S2).

We describe the design as pre-specified rather than pre-registered, and set out the deposit history so that readers can judge what that means here. A protocol stating the study design, the five query domains, the outcome definitions, and the intended taxonomy was deposited on the Open Science Framework on 27 May 2026 (DOI 10.17605/OSF.IO/DK2PM). The first queries were issued on 26 May 2026, so the deposit followed the opening day of an eight-day collection window rather than preceding it. The item-level benchmark, raw responses, and coded dataset were deposited on completion of coding in July 2026. No hypothesis, outcome definition, or analytic decision was altered after the deposit, and the deposited protocol can be compared against what is reported here; but the design does not meet the standard of a formal prospective registration, and we do not claim it does.

The severity stratification reported here was specified after the primary analysis and is presented as a secondary, exploratory reanalysis.

### 2.2. Models and Query Parameters

Five models were queried through official APIs between 26 May and 2 June 2026: GPT-4o (gpt-4o-2024-11-20), Claude Opus 4.5 (claude-opus-4-5), Gemini 2.5 Flash (gemini-2.5-flash), DeepSeek-V3 (deepseek-chat), and Llama 3.3 70B Instruct (meta-llama/Llama-3.3-70B-Instruct-Turbo) via Together AI. Only three parameters were set on any call: the model identifier, temperature = 0.0, and max_tokens = 4000, together with a single user message carrying that item’s prompt. No seed was supplied to any provider, so run-to-run variation is unconstrained, and determinism should not be inferred from temperature = 0 alone. No system prompt, tool, retrieval option, or browsing capability was supplied, so responses reflect parametric knowledge under a fixed prompt. Each query was issued as an independent, stateless request: no conversation history or session state was carried between items, and a fresh client was instantiated per provider. Failed calls were retried up to three times with a backoff of five seconds multiplied by the attempt number, and the dispatch code raises rather than substituting a partial response if all attempts fail. No response reached the token limit, so no output was truncated. Findings apply to these snapshot versions and parameters. The per-provider request payloads, endpoints, model identifiers, prompt template, and response-handling code are in the deposited repository (query_runner.py and config.py), where these settings can be checked directly.

### 2.3. Query Benchmark

One hundred standardised items spanned five domains of twenty items each: clinical protocols (A), materials and appliances (B), cephalometric and biomechanical concepts (C), cases and landmark studies (D), and adversarial items (E). Three orthodontists not otherwise involved rated each item for relevance and clarity; the scale-level content validity index (average) was 0.923 (Table S1).

Forty-five items contained a fabricated element. Twenty comprised domain E, in which the fabricated entity was the subject of the question. Twenty-five were embedded within domains B, C, and D as realistic clinical questions containing a fabricated detail—a non-existent bracket system named among real ones, a fictitious cephalometric index, and a fabricated reference attributed to a real author. The remaining 55 items contained no fabricated element. The benchmark is therefore a stress test of susceptibility to false premises, not a representative sample of clinical queries, and the resulting rates should not be read as the frequency with which these models hallucinate in ordinary use. Results are reported for the three strata separately—natural items, embedded false premises, explicitly adversarial items—so that the contribution of each is visible.

Twenty-six fabrication types were used across the 45 items (Table S2). Five types account for 24 of them: fabricated concepts 9, products 8, citations 3, persons 2, and cases 2. The remaining 21 items carry one type each, covering fabricated journals, conferences, classifications, equations, landmarks, materials, societies, documents, histories, biomarkers, software, protocols, measurements, awards, techniques, surveys, books, and trials, together with a real product described with fabricated detail, a combined product, and a metacognition probe. The 100 items are a purposively assembled benchmark rather than a probabilistic sample from a defined population of orthodontic queries, and no such sampling frame exists. Inferential statistics here describe variation across the five models on this fixed item set; they do not licence extrapolation to the wider universe of clinical prompts. Effect estimates, confidence intervals and the composition of the benchmark therefore carry more weight than the *p*-values, and replication on an independently constructed item set is the appropriate test of generalisability.

Ground truth is fixed by construction for these 45 items only, and it is worth stating which outcomes rest on what. H2, H3, and H4 are deterministic for those items: an entity that does not exist cannot be correctly elaborated, so any substantive elaboration is unsupported by design. H1 rests on bibliographic retrieval and inherits the coverage of the databases queried. H5 rests on a regular-expression rule applied to a fixed list of four superseded assertions, and fired on one response, so it carries almost no weight in the totals. H6 is flagged by rule and then adjudicated by two orthodontists, so it rests on expert clinical judgement. The severity tier to which each category is assigned rests on an author-defined mapping and is not fixed by construction at all.

### 2.4. Citation Verification

Citations were extracted and queried against the PubMed E-utilities [16] and CrossRef [17] APIs. A DOI was resolved directly against CrossRef; a PMID against PubMed efetch; an author–date reference through a two-stage PubMed search, first on author, year, and a title fragment, then on the title alone. Three outcomes follow. A citation is verified when a record was retrieved. It is non-retrievable when every applicable route is completed and returns no record. It is not assessed when the request itself failed at the transport layer, so retrieval was never completed. We do not equate non-retrieval with fabrication as a matter of definition, since a reference may exist in a source these APIs do not index. To establish how far the two coincide in practice, we carried out a manual check of the subset in which the automated pipeline verified nothing at all. Sixty-nine responses contained citations but yielded no verified citation. Every DOI appearing in them—143 distinct identifiers—was queried independently against the CrossRef REST API and the DOI resolver. An identifier was recorded as absent only when both returned a definite 404; any transport error would have been recorded as indeterminate. The procedure was validated against five DOIs known to exist, all of which resolved. Two definitions of a citation hallucination are therefore reported: a permissive one counting every citation not verified, which is the convention in the literature we compare against, and a strict one counting only non-retrievable citations. The strict definition is the more defensible; the permissive one is retained for comparability and reported alongside. The category is labelled unverifiable citations rather than fabricated citations for the same reason; the column name in the deposited dataset is unchanged, so the two can be matched.

Verification was run once over the full response set after collection closed, with a minimum interval of 0.35 s between PubMed calls where an NCBI API key was in use and 0.5 s otherwise. The author–date search used the first author’s surname, the year, and the first six words of the title as a phrase-restricted title field, returning at most five candidates; if none matched, a second pass searched the title fragment alone and the result was marked as a title-only match. Matching was exact on DOI and PMID and phrase-based on title; no fuzzy threshold was applied, so orthographic variants and altered titles would fail the first pass and be caught, if at all, by the second. Preprints, monographs, clinical guidelines, non-indexed journals, and online-ahead-of-print records are not reliably covered by either database, and a citation to any of them would be recorded as non-retrievable although it exists; this is the principal reason we do not equate non-retrieval with fabrication. Verification ran on extracted citation strings alone, with no model identifier in the request, so it was blind to model identity by construction. Duplicate records were resolved by taking the first returned candidate, and partial matches were accepted only when the retrieved record carried the same first author and year.

### 2.5. Taxonomy and Severity Tiers

Responses were coded with a seven-category orthodontics-specific taxonomy: unverifiable citations (H1), fabricated appliances or devices (H2), fabricated clinical protocols (H3), misattributed discoveries (H4), anachronistic clinical knowledge (H5), pseudo-quantitative claims (H6), and metacognitive failure, defined as absence of epistemic hedging on an item with a fabricated premise (H7). Operational criteria as implemented are given in Table S3.

Categories were assigned to tiers by the action a clinician acting on the content would take (Table 1). The mapping is exploratory and author-defined. It was fixed before any tier-level analysis and is reported in full so that readers can apply a different boundary, but it has not been externally validated, was not derived from any published patient-safety instrument, and should not be read as one. Placing H1 in the epistemic tier, for instance, is one defensible choice rather than an objective fact: a clinician who cannot trace a reference is impeded, not misled about treatment, but a reader who weighs traceability more heavily would order the tiers differently. The sensitivity analysis in Section 3.5 bounds how much the choice matters. H7 is not a hallucination category at all but a property of how a response is expressed, and is analysed as a modifier rather than as one of the seven content types.

**Table 1:** Assignment of taxonomy categories to severity tiers, with a representative example, how each category was detected, and which other categories it can co-occur with. The mapping is exploratory and author-defined; it was fixed before any tier-level analysis. Because categories co-occur, the tier counts in Table 2 are not mutually exclusive.

Tier	Categories	Definition	Representative Example	Detection	Can Co-Occur with
Tier 1—clinical	H3, H5	Could alter a treatment decision. A fabricated protocol or a superseded clinical claim, if acted upon, changes what is done to the patient.	A response elaborating in detail on a named clinical protocol that does not exist, presented without qualification.	H3 automated (fabricated-element item + substantive response + no hedge); H5 automated (regular-expression match); adjudicated numeric errors human-rated.	H1, H2, H4, H6, H7
Tier 2—operational	H2	Could cause futile procurement or workflow expenditure. A fabricated appliance costs clinician time but does not itself alter a treatment decision.	A response describing the specifications and indications of a bracket system that does not exist.	Automated (fabricated-element item + substantive response + no hedge).	H1, H3, H4, H6, H7
Tier 3—epistemic	H1, H4	Impedes verification without altering the clinical recommendation.	A citation that cannot be retrieved from CrossRef or PubMed, or a real finding attributed to the wrong author or trial.	H1 automated (API verification); H4 automated (fabricated-element item + no hedge).	all other categories
Adjudicated	H6	Numeric claims without verified support. Automated coding cannot distinguish an incorrect value from a correct but unsourced one; adjudicated separately and promoted to Tier 1 when judged incorrect.	A force value or prevalence figure stated without any verifiable source.	Flagged automatically (two or more numeric claims with no verified citation, or three or more with under 30% of citations verified); each flagged response then rated by two orthodontists.	all other categories
Modifier	H7	Absence of hedging. Not a content category.	A declarative answer to a question containing a fabricated premise, with no expression of uncertainty.	Automated (fabricated-element item + zero hedge markers from a 36-pattern set).	H1–H6

Two structural constraints follow from the coding rules and bear on interpretation. H2–H4 and H7 fire only on items carrying a fabricated element, so Tier 1 membership is largely confined to those items by construction. H3 also requires the absence of epistemic hedging, so hedged responses cannot enter Tier 1 through H3. Analyses below are structured to separate what these constraints determine from what they do not.

**Table 2:** Severity distribution across 500 responses. Tiers are not mutually exclusive.

Severity Tier	*n*	%	95% CI
Tier 1—clinically consequential	65	13.0	10.3–16.2
Tier 2—operationally consequential	98	19.6	16.4–23.3
Tier 3—epistemically consequential	420	84.0	80.5–87.0
Any hallucination (permissive)	449	89.8	86.8–92.2
Any hallucination (strict)	371	74.2	70.2–77.8

### 2.6. Reliability

Coding provenance differs by category and bears on what reliability statistics mean here. H1, H5, and the H6 flag were produced by deterministic rules with no human judgement, so inter-rater reliability does not apply to them. H1 fires when at least one extracted citation is not verified, which is the permissive criterion. H2, H3, and H4 fire only when the response to a fabricated-element item is substantive, defined as at least 500 characters, and carries no hedge marker; the fabrication type determines which of the three applies, and some types map to more than one, so a single response can carry several. H7 requires only a fabricated-element item and the absence of a hedge marker, with no length condition, which is why it fires far more often than the content categories. H5 matches a fixed list of four regular expressions covering superseded clinical assertions, and fires on a single response. The H6 flag has two branches: it fires when a response contains two or more unsupported numeric claims and no verified citation, or three or more such claims with fewer than 30% of its citations verified. Of the 53 flagged responses, 27 came from the first branch and 26 from the second.

H2, H3, H4, and H7 required human judgement and were double-coded on a 10% subsample. That subsample comprised 50 responses, drawn as ten benchmark items across all five models so that every model contributed equally. Because κ is unstable where an event is rare, we report raw agreement and category prevalence alongside it. Agreement was 50 of 50 for H2, H3, and H4, with subsample prevalence of 6%, 12%, and 8%, respectively, and 44 of 50 for H7, with prevalence 54%. The three κ values of 1.00 should therefore be read as the absence of any observed disagreement on categories that fired between three and six times. They are not evidence of reliability that would survive a larger or more balanced sample. H7, the only category occurring often enough for κ to be informative, returned 0.76 (95% CI 0.58–0.94). Coders worked from response text with the model identifier removed, so double-coding was blind to model identity, and the automated primary coding is blind by construction. Disagreements were not reconciled: they arose only on H7, and both raters’ assignments are reported rather than merged. Coding was per response rather than per claim: a category fires once for a response irrespective of how many instances it contains, so the counts are of responses carrying a category, not of individual unsupported claims.

For the adjudication of unsupported numeric claims, both authors independently rated all 53 flagged responses, blinded to model identity and query identifier and without access to each other’s ratings. The rating instruction, fixed before adjudication and deposited with the study materials, defined the reference standard as current orthodontic evidence and directed raters to treat any value falling inside a debated range as acceptable rather than incorrect. No fixed numeric tolerance was imposed, because the quantities involved are heterogeneous: a force magnitude, a scoring threshold, and a prevalence estimate do not share a common scale. The operative criterion was whether the stated value falls within the interval that current orthodontic sources would support for that quantity, and the interval the rater applied is recorded for every verdict of incorrect in Table S5. A claim was judged incorrect only where one of three conditions held: it contradicted the published specification of a named instrument or protocol; it asserted a single universal figure for a quantity that varies by population, index, or age group; or it misapplied a statistical convention. Every verdict of incorrect required the rater to state the correct value or range, and those statements are reported in full in Table S5, so the standard applied to each item is inspectable rather than assumed. Three-level agreement was 90.6% (48 of 53), κ = 0.81 (95% CI 0.65–0.97). All five disagreements lay on the boundary between “acceptable but unsourced” and “indeterminate”; none concerned whether a value was incorrect. Agreement on the incorrect-versus-not distinction, which alone determines promotion to Tier 1, was complete (53 of 53, κ = 1.00). Disagreements were not resolved by discussion: both raters’ independent verdicts are reported. This was an investigator assessment, not external validation.

### 2.7. Statistical Analysis

Proportions are reported with Wilson intervals [20], using the Jeffreys interval where k = n [21]. These are descriptive intervals for a single proportion and do not account for clustering by benchmark item; they describe the precision of each rate on this item set rather than supporting inference to a wider population. Where an interval carries an inferential claim about a difference between models, it is computed on discordant pairs and identified as such.

Because every model answered the same 100 items, responses are matched at item level and are not independent; analyses that compare models therefore use methods for matched or clustered binary data. The omnibus comparison across the five models uses Cochran’s Q, followed where relevant by pairwise McNemar tests with Holm adjustment. The same combination has been applied to model comparisons on a shared orthodontic case set [22], where the paired structure was handled explicitly for the same reason. Comparisons in which items rather than models form the groups use generalised estimating equations with an exchangeable working correlation and the item as the clustering unit. To quantify how much clustering matters for each outcome, we also fitted a logistic mixed model with a random item intercept and report the implied intra-class correlation. Two comparisons are defined at item level rather than response level: the domain contrast and the model × domain interaction. For the first, we used Kruskal–Wallis. For the second, we used a permutation test that shuffles domain labels across items. That design preserves each model’s overall rate and each item’s difficulty, and stays valid when cells are empty. Pearson χ2 values are given alongside for comparison with the published literature, which has generally treated such responses as independent; where the two disagree, the matched estimate is the one we interpret. Analyses used Python 3.11.9 (pandas 2.2.2, scipy 1.13.1, statsmodels 0.14.2); code is available at https://github.com/drmustafaozcan/llm-ortho-hallucination (accessed on 27 September 2026).

The sample was fixed by the deposited benchmark (100 items × 5 models) and was not sized for the severity analysis reported here. Power was estimated on the matched design rather than on an unpaired comparison, since the latter would misstate it in both directions. For a paired binary contrast, the relevant quantity is the proportion of items on which two models disagree, which was 0.06 at the clinical tier. Simulating the exact McNemar test at two-sided α = 0.05 under that discordance, 100 items give 56% power to detect the 6-point difference actually observed, 83% power for an 8-point difference, and 94% for a 10-point difference; roughly 130 items would be needed for 80% power at 6 points. A null or borderline result at the clinical tier is therefore consistent with a real difference in the size observed, and does not count as evidence against one.

## 3. Results

### 3.1. Severity Distribution

Of 500 responses, 449 (89.8%; 95% CI 86.8–92.2) contained at least one hallucination under the permissive definition. The strict citation criterion reduced this to 371 (74.2%; 95% CI 70.2–77.8). Three quantities are easily conflated here, so we separate them. Three hundred and sixty-four responses contained at least one citation that could not be assessed. Of these, 147 contained no non-retrievable citation, so their citation record consisted only of verified and not-assessed entries. Seventy-eight responses—the difference between the permissive and strict counts—were classified as hallucinated under the permissive definition solely because a citation could not be assessed, with no other category firing. It is the last of these, 78, that the permissive-versus-strict contrast turns on.

Sixty-nine of the 457 responses containing at least one citation yielded no verified citation. Manual checking of every DOI in those responses returned the same verdict as the pipeline in all 143 cases: none was registered with CrossRef and none resolved through the DOI system. Non-retrieval by the automated pipeline therefore corresponded to absence from both registries wherever we were able to check it directly, although the caution stated in Section 2.4 continues to apply to citations lacking a DOI, which this check could not cover.

Of the 53 responses carrying unsupported numeric claims, adjudication classified 9 (17.0%) as containing an incorrect value, 36 (67.9%) as acceptable but unsourced, and 8 (15.1%) as indeterminate (Table S5). The automated flag therefore over-identified numeric error by roughly five to one: 44 of the 53 flagged responses were not judged incorrect. The nine judged incorrect were promoted to Tier 1.

Tier 1 therefore comprised 57 responses carrying a fabricated protocol (H3), one anachronistic clinical claim (H5), and seven adjudicated numeric errors not co-occurring with either, with the remaining two adjudicated errors already counted under H3. Stratified by consequence, 65 responses (13.0%; 95% CI 10.3–16.2) were clinically consequential, 98 (19.6%) operationally consequential, and 420 (84.0%) epistemically consequential (Table 2). Because a single response may carry categories belonging to more than one tier—a fabricated protocol accompanied by a fabricated citation, for instance—the three-tier counts are not mutually exclusive and sum to more than 500. Assigning each response to the highest tier for which it qualifies resolves the overlap; that composition is shown in the next section.

### 3.2. Between-Model Differences Contract Sharply at the Clinical Tier

**Figure 1:**
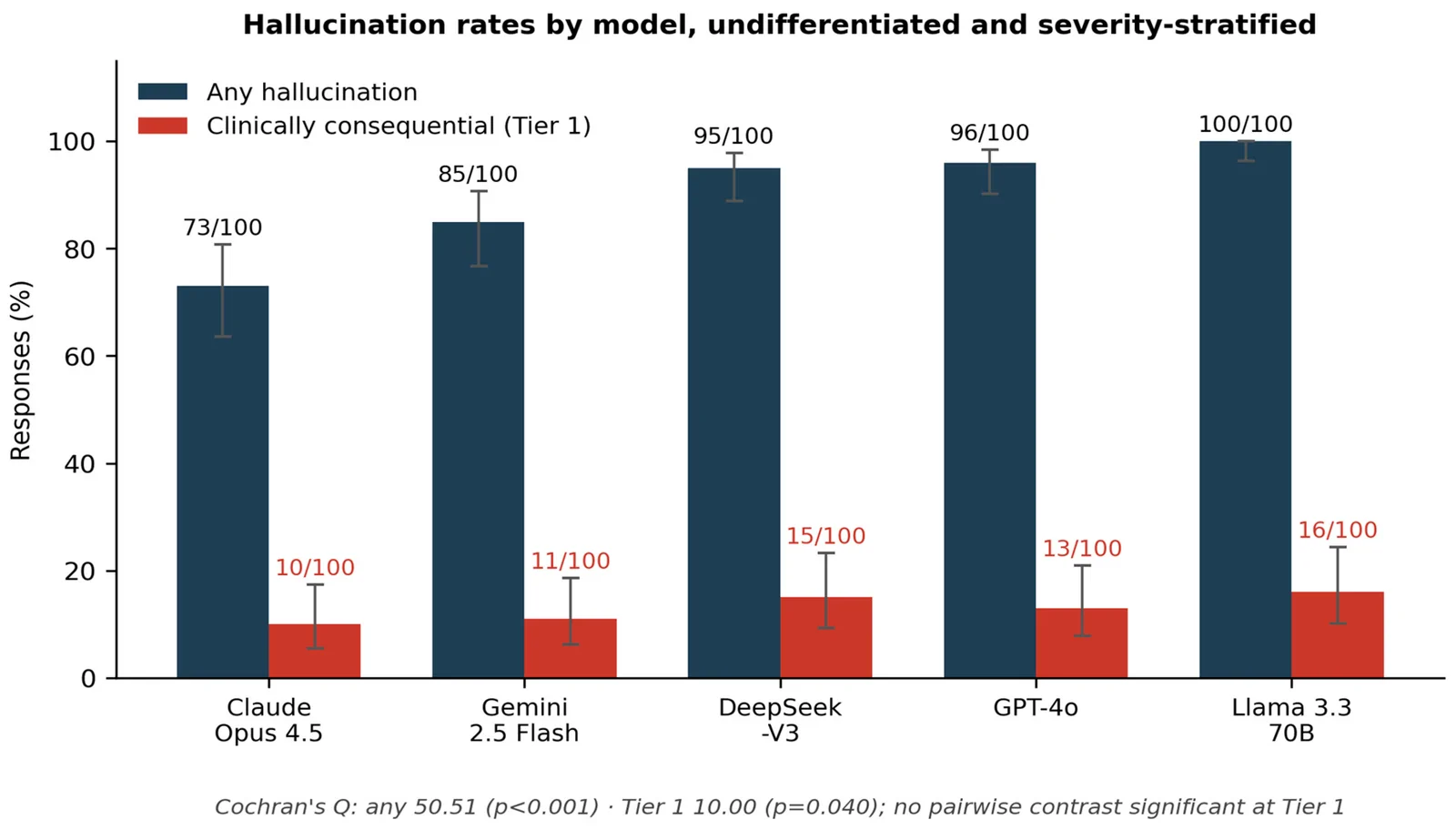
Hallucination rates by model, undifferentiated and restricted to the clinically consequential tier, with Wilson 95% confidence intervals and counts out of 100 responses per model. “Clinically consequential” denotes assignment to Tier 1 of the author-defined taxonomy, not an observed clinical decision or outcome. Between-model comparison uses Cochran’s Q, which accounts for the matched item structure.

**Figure 2:**
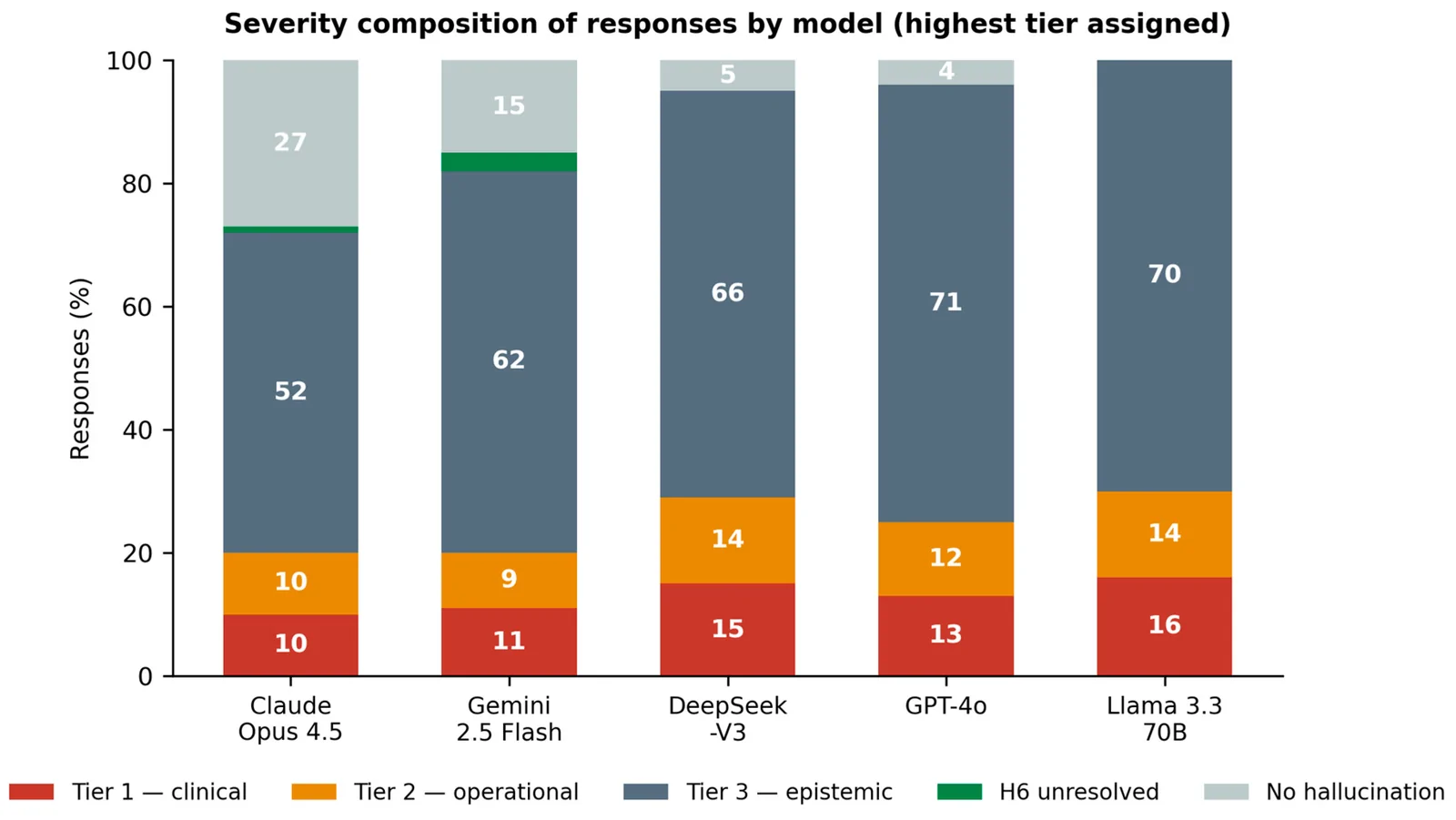
Severity composition of responses by model under the highest-applicable-tier rule: each response is counted once, under the highest tier for which it qualifies, so the bars sum to 100% within each model. This differs from Table 2, which reports tier prevalence without that rule and therefore sums to more than 100%; the two summaries answer different questions and should not be read against each other. Tier labels denote author-defined categories rather than observed clinical consequences.

Undifferentiated rates ranged from 73% to 100%. Under the matched analysis, this difference was large and highly significant (Cochran’s Q = 50.51, df = 4, *p* < 0.001; Pearson χ^2^ = 51.84 for comparison), and four of the ten pairwise contrasts survived Holm adjustment. Tier 1 rates ranged from 10% to 16%. Here the matched omnibus test was borderline: Cochran’s Q = 10.00, df = 4, *p* = 0.040, with a GEE Wald χ^2^ of 9.41, df = 4, *p* = 0.052. No pairwise contrast survived Holm adjustment. The largest was Claude Opus 4.5 against Llama 3.3, with 6 versus 0 discordant items, unadjusted *p* = 0.031 and adjusted *p* = 0.313. The naive Pearson test would have returned χ^2^ = 2.30, df = 4, *p* = 0.681 here; we report it only to show how far an independence assumption misleads on this outcome (Table 3, Figure 1). The severity composition underlying these rates, with each response assigned to its highest applicable tier, is shown in Figure 2.

Two features explain why the same five models behave so differently on the two outcomes. First, item clustering differs sharply between them. For any hallucination, the intra-class correlation is 0.07, so matched and unmatched analyses agree. For Tier 1, it is 0.83, indicating strong item-level heterogeneity: responses to the same item resemble one another far more than responses to different items do. We report this as a property of the clustering rather than as a variance decomposition between item and model, since the intra-class correlation from a random-intercept model quantifies item-level clustering on the latent scale and does not partition variance against a fixed effect. Part of the heterogeneity is also structural, because the category accounting for most Tier 1 events can fire only on items carrying a fabricated element. Second, the design was not sized to detect small between-model differences. Estimated on the matched design, the 100 items gave 56% power to detect the 6-point gap actually observed and 83% power for an 8-point gap; about 130 items would have been required for 80% power at 6 points. The matched difference between the two extreme models was 6.0 points (unadjusted 95% CI 1.3 to 10.7), computed on discordant pairs rather than on independent proportions. The interval is not adjusted for multiplicity and should be read alongside the Holm-adjusted pairwise test for that contrast, which was not significant (adjusted *p* = 0.313); the two are not in conflict, since an unadjusted interval and a multiplicity-controlled test answer different questions. We therefore do not claim the models are equivalent in clinical safety. What the data support is narrower: the 27-point separation seen on undifferentiated prevalence contracts to 6 points at the clinical tier, the omnibus test falls from Q = 50.51 to Q = 10.00, and no individual pair of models can be distinguished once multiplicity is controlled.

The ranking obtained from undifferentiated rates therefore reflects citation behaviour. Llama 3.3 generated 10.8 citations per response, 48.9% of them non-retrievable, against 3.0 per response and 15.8% for Claude Opus 4.5 (Table S6)—a difference that propagates into aggregate prevalence while leaving clinically consequential rates far closer together, with no pairwise contrast surviving adjustment.

Because the citation criterion drives that ranking, we report it per model. Under the permissive definition, the rates are 73%, 85%, 95%, 96%, and 100% for Claude Opus 4.5, Gemini 2.5 Flash, DeepSeek-V3, GPT-4o, and Llama 3.3, respectively; under the strict definition they fall to 50%, 64%, 79%, 78%, and 100%. The proportion of citations that could not be assessed because the API request failed was 23.8%, 43.2%, 33.1%, 30.6%, and 28.9%, and the proportion confirmed non-retrievable was 15.8%, 23.3%, 23.1%, 32.0%, and 48.9% (Table S6). The rank order is almost unchanged between the two definitions (Spearman ρ = 0.90; DeepSeek-V3 and GPT-4o exchange places), and the between-model difference remains large under the strict definition (Cochran’s Q = 82.41, df = 4, *p* < 0.001). The choice of criterion therefore changes the absolute prevalence substantially but not the ordering.

### 3.3. Clinically Consequential Output Followed False Premises

**Figure 3:**
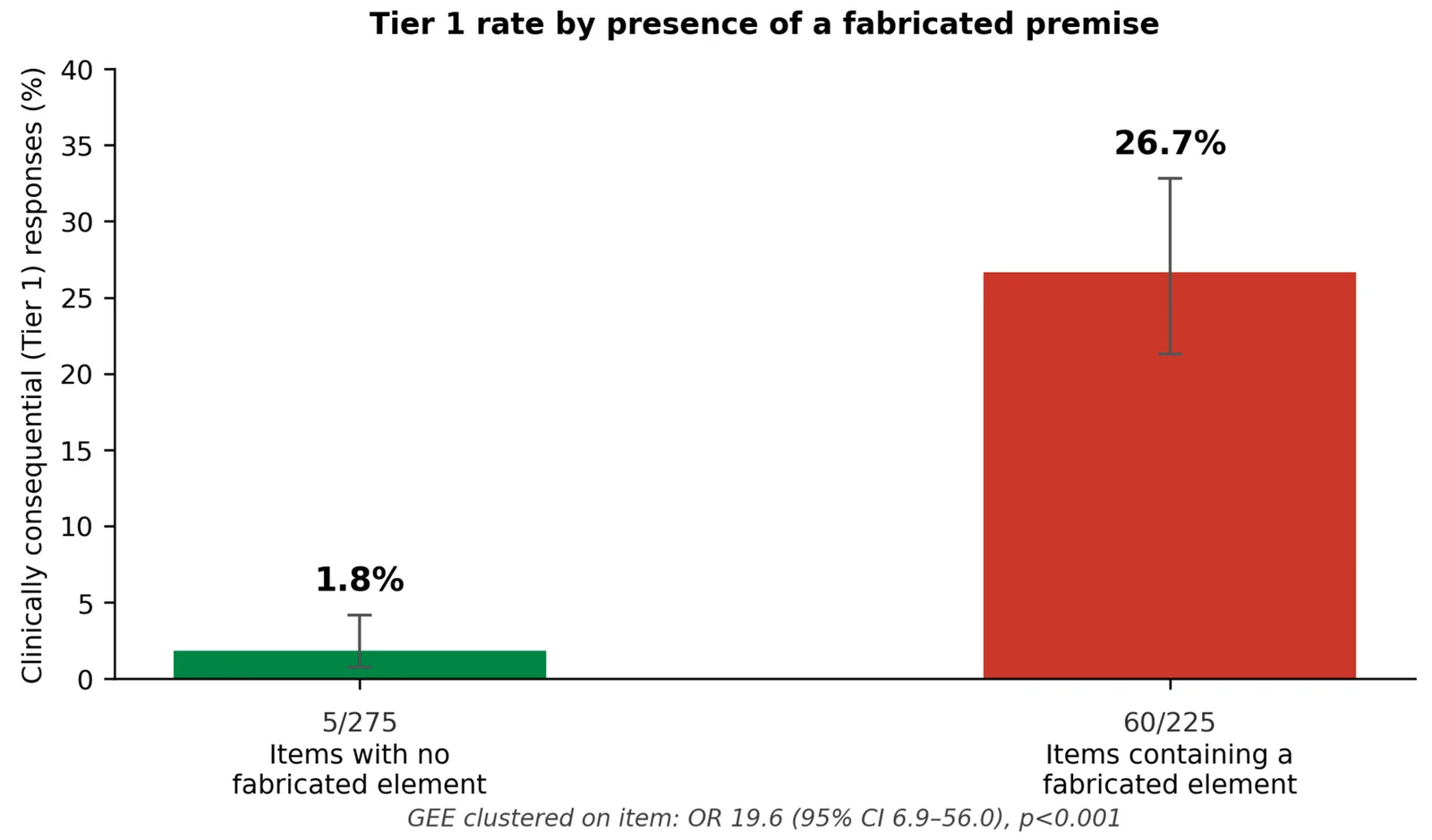
Tier 1 rate for responses to items containing a fabricated element versus items without one, with Wilson 95% confidence intervals and counts beneath each bar. Denominators differ (225 versus 275). The contrast is partly determined by the coding rules, since the tier-defining category can fire only on items carrying a fabricated element, and is presented as a descriptive property of the taxonomy rather than an unbiased estimate of differential clinical risk.

Tier 1 occurred in 60 of 225 responses to items containing a fabricated element (26.7%; 95% CI 21.3–32.8) and in 5 of 275 responses to items without one (1.8%; 95% CI 0.8–4.2). Under a GEE model clustered on item, the odds ratio was 19.64 (95% CI 6.89–55.98; *p* < 0.001; Figure 3). Of the five, four were adjudicated numeric errors, and one was an anachronistic clinical claim; no response to an item without a fabricated element met the criteria for a fabricated protocol. Separating the fabricated-element items into the two strata defined in Section 2.3, Tier 1 occurred in 31 of 125 responses to items with an embedded false premise (24.8%; 18.1–33.0) and 29 of 100 responses to explicitly adversarial items (29.0%; 21.0–38.5). Against natural items, the odds ratio was 17.81 (5.34–59.36) for embedded items and 22.06 (6.50–74.89) for explicitly adversarial ones (GEE clustered on item; Wald χ^2^ = 31.26, df = 2, *p* < 0.001). Embedded and explicitly adversarial items behaved almost identically, so the effect is not an artefact of the obviousness of the adversarial block.

This asymmetry is partly structural, since H3 can fire only on items carrying a fabricated element, and we do not present it as evidence that models never invent protocols spontaneously. The constraint is a reason to seek external corroboration rather than to discount the contrast. A response-level rating by clinicians blind to model identity, applied to the same responses, would establish whether the severity ordering we impose by rule matches the ordering clinicians would assign by judgement. It would do so independently of the coding rules that produce this asymmetry. Nor do we claim a causal mechanism. What the data establish is an association within this benchmark: clinically consequential output was concentrated in responses to items whose premise was false, and was rare elsewhere. Whether models would generate such content unprompted in ordinary clinical use is a different question that this design cannot answer.

### 3.4. Severity Depended on What Was Fabricated

**Figure 4:**
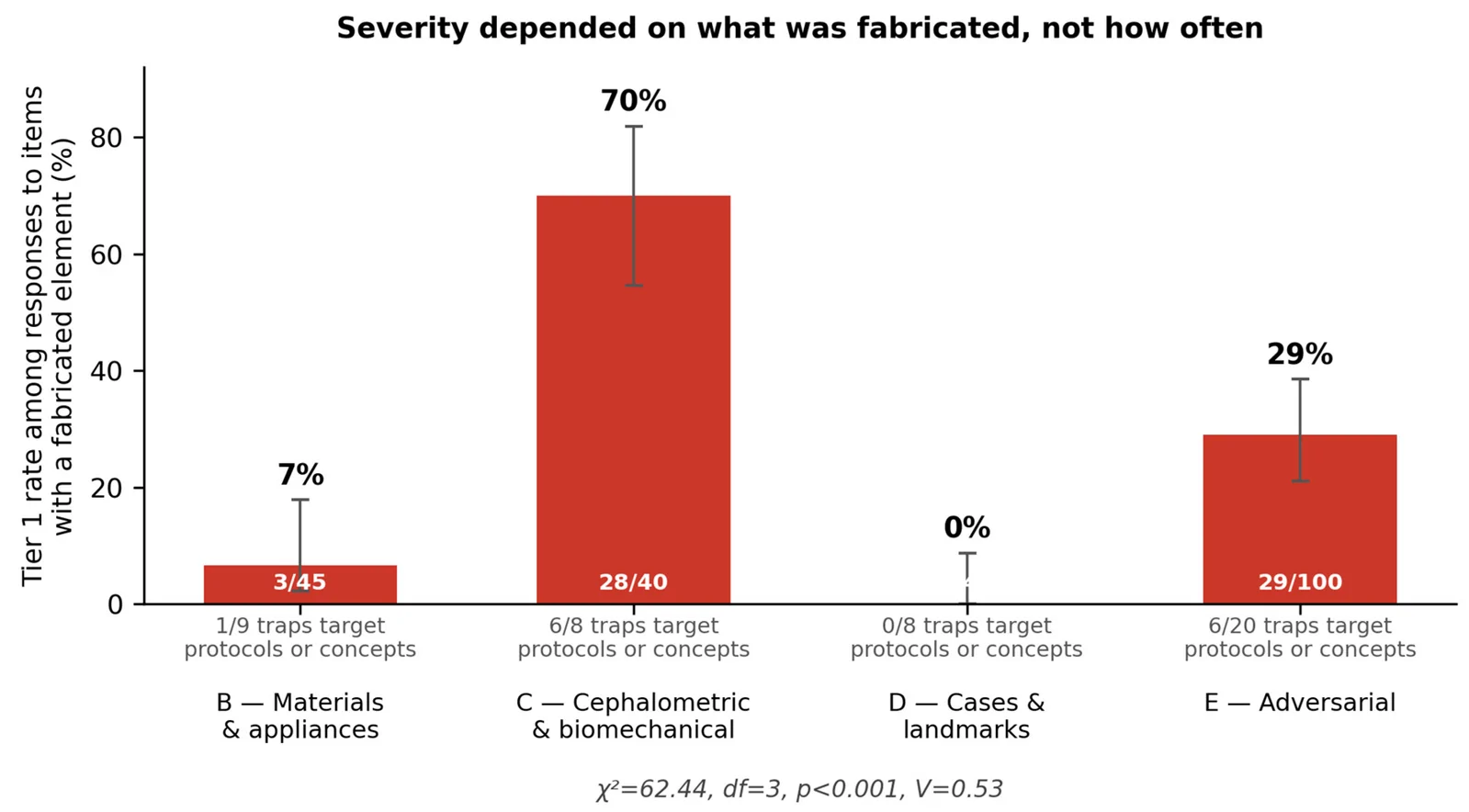
Tier 1 rates among responses to items containing a fabricated element, by query domain, with Wilson 95% confidence intervals. Denominators differ by domain (45, 40, 40 and 100 responses). The gradient is partly taxonomy-driven, since protocols and concepts map to the tier-defining category while citations and attributions map to the epistemic tier; it is therefore descriptive of the classification scheme rather than an unbiased domain comparison. The formal test is at item level (Kruskal–Wallis, Section 3.4).

Restricting analysis to the 225 responses to fabricated-element items holds the structural constraint constant. Within this subset, Tier 1 rates differed sharply by domain: 70% for cephalometric and biomechanical items, 29% for adversarial items, 7% for materials and appliances, and 0% for cases and landmark studies (Table 4, Figure 4). Domain is a property of the item rather than of the response, and one domain produced no Tier 1 output at all, which makes a logistic model of this contrast separable and its Wald statistic uninterpretable. We therefore tested it at the item level, taking as the outcome the proportion of the five models producing Tier 1 output on each item. This treats each item once, requires no assumption about within-item correlation, and is unaffected by the empty cell: Kruskal–Wallis H = 13.92, df = 3, *p* = 0.003, ε^2^ = 0.27.

The gradient tracks what was fabricated rather than how many fabrications a domain contained. Domains C and D contained a similar number of fabricated-element items (eight each) but differed completely in outcome. In domain C, six of eight fabricated entities were protocols, concepts, or equations, which map to Tier 1; in domain D, none were, all being unverifiable citations, people, or trials, which map to Tier 3. The severity of a model’s failure was thus determined by the category of the false premise, not by its frequency. We also asked whether the relative standing of the models varies by domain. Maximum-likelihood estimation of a model × domain interaction is degenerate here because the cases-and-landmarks domain produced no Tier 1 response for any model, so we tested the interaction by permutation instead, shuffling domain labels across items in a way that preserves each model’s overall rate and each item’s difficulty. There was no evidence of interaction (observed interaction sum of squares 0.027; permutation *p* = 0.789, 10,000 permutations); the same test applied to undifferentiated hallucination, where between-model differences are large, returned *p* = 0.068, indicating that it is capable of detecting an interaction when one is present. The rank order of the models was also similar in the two domains carrying most of the Tier 1 output (Spearman ρ = 0.89 between cephalometric and adversarial items). Every model showed the same domain ordering, with cephalometric items highest and cases-and-landmarks items empty.

### 3.5. Sensitivity of the Severity Threshold

The tier mapping was assigned by the authors rather than validated externally. We therefore recomputed the clinical tier under six definitions spanning a range of boundaries that could reasonably be drawn, from fabricated protocols alone to a maximal definition treating every category except pure citation error as clinically consequential (Table 5).

The gap between undifferentiated and clinically consequential prevalence persisted across every definition, narrowing from 7.9-fold to 2.1-fold but never closing. Under the matched test, the between-model omnibus was non-significant only under the H3 + H5 definition (Q = 5.75, *p* = 0.219), and borderline under the two narrowest (Q = 9.43, *p* = 0.051 and Q = 10.00, *p* = 0.040). It becomes significant once H2, H6, or H7 are folded in. The contraction of the between-model difference is therefore a property of the narrow, clinically defined tiers rather than of the taxonomy as a whole, and the prevalence gap itself is not an artefact of where the threshold was drawn.

Response length is a plausible confounder for these findings. Median length differed markedly between models, from 3335 characters for Claude Opus 4.5 to 5910 for Gemini 2.5 Flash (Kruskal–Wallis H = 198.0, *p* < 0.001), and length enters the coding rules directly, since H2, H3, and H4 require a substantive response. It does not, however, predict Tier 1 output once the presence of a fabricated element is accounted for (GEE clustered on item: odds ratio 1.03 per 1000 characters, 95% CI 0.99–1.06, *p* = 0.150), nor within fabricated-element items alone (1.04, 0.96–1.12, *p* = 0.364). The 500-character threshold is in practice not binding either: between 94% and 100% of responses cleared it in every model. Verbosity therefore does not explain the severity findings, although it does contribute to the citation counts on which undifferentiated prevalence depends.

A separate sensitivity analysis addresses the hedging condition inside H3. As specified, H3 fires only when a response to an item of the relevant fabrication type is substantive and carries no epistemic hedge, which makes linguistic framing part of a content-severity definition. A fabricated protocol may of course be clinically consequential even when introduced with a qualifier, so we recomputed the clinical tier with that condition relaxed. Sixty-five responses met the other two conditions; 57 carried no hedge, four carried one hedge marker, and four carried two or more. Tolerating a single hedge raises Tier 1 from 13.0% to 13.8% (95% CI 11.1–17.1); disregarding hedging altogether raises it to 14.4% (11.6–17.7). The prevalence gap is therefore insensitive to this rule, narrowing only from 6.9-fold to 6.2-fold. The between-model omnibus is more sensitive, moving from Q = 10.00 (*p* = 0.040) to Q = 4.00 (*p* = 0.406), which reinforces rather than weakens our reading that between-model differences at this tier are not robust. We retain the hedging condition as primary because removing it entirely would let the category fire on any substantive answer to a fabricated premise, measuring response length rather than failure to reject the premise. The choice makes little difference to the quantity the paper is about, and we report both.

### 3.6. Epistemic Hedging

Hedging frequency on fabricated-element items differed between models, from 42.2% (Claude Opus 4.5) to 8.9% (Llama 3.3) (Cochran’s Q = 22.86, df = 4, *p* < 0.001). Tier 1 occurred in 1 of 50 hedged responses and 59 of 175 unhedged responses. We do not report this contrast as an association, because H3 requires the absence of hedging by definition and therefore forces most of it. Testing the relationship on the only Tier 1 component that carries no hedging condition—the adjudicated numeric errors—showed no association. Seven of the nine adjudicated errors qualify for this test; the remaining two co-occurred with H3 and therefore inherit its hedging condition. Across all 500 responses, these seven were distributed 1 of 57 hedged versus 6 of 443 unhedged (Fisher *p* = 0.574). The denominator here is the full sample rather than the 225 fabricated-element responses, because adjudicated numeric errors are not confined to fabricated-element items. On the available hedge-independent evidence, hedging does not predict clinical consequence. We report this analysis rather than omitting it because the failure it illustrates is general. Hedging entered H3 as a defining condition and then reappeared as the predictor in a test of that same category, which guarantees a strong association whatever the models actually do. Any coding scheme that builds a variable into an outcome definition forfeits the ability to test that variable against the outcome, and the only informative test is the one restricted to a component the definition does not constrain. We suggest that severity taxonomies keep content categories and expression modifiers separate for this reason, and we have reclassified H7 accordingly.

## 4. Discussion

### 4.1. Prevalence and Clinical Consequence Diverge

Stratifying by clinical consequence changed both the magnitude of the problem and its interpretation. Prevalence of 89.8% fell to 13.0% when restricted to content the authors classified as capable of altering a treatment decision—roughly a sevenfold difference, and a gap that persisted from 7.9-fold to 2.1-fold across every alternative severity definition examined in the sensitivity analysis (Table 5). Aggregate figures in this literature therefore exceed the rate of potentially consequential content by a large and quantifiable margin. Whether that gap corresponds to a gap in realised clinical risk is a further question this design does not address, since no clinician acted on any output and no patient was exposed to one.

### 4.2. What the Model Comparison Does and Does Not Show

The contraction of between-model differences at the clinical tier is the more consequential result. Models separated by 27 percentage points on undifferentiated prevalence were separated by 6 points at the clinical tier, where the omnibus test was borderline and no pairwise contrast survived adjustment for multiplicity. We do not read this as equivalence: the study was not sized to detect a difference in that size, and a borderline omnibus with no surviving pairwise contrast is weak evidence in either direction. A meta-analysis of 39 orthodontic patient-education studies points the same way from a different direction: pooled across instruments, ChatGPT-4-class models and Gemini did not differ on rated accuracy (mean difference −0.12, 95% CI −0.42 to 0.17), and no accuracy or quality pool reached significance under either pooling method [23]. What our data show is that the two measures rank models differently and that the apparent separation on prevalence is driven largely by citation behaviour—a model that cites sparingly and accurately will appear safer than one that cites profusely, irrespective of the clinical soundness of either. This matters wherever such rankings inform practice. Procurement decisions, institutional guidance, and the selection of a model for a decision-support pipeline all rest on comparative reliability figures. A figure that separates models by 27 points on citation behaviour, while leaving them barely distinguishable on clinically consequential error, does not support the choice it appears to justify.

### 4.3. Where Clinically Consequential Output Arose

The concentration of clinically consequential output in responses to items with a false premise is consistent with the pattern Martina and colleagues observed from a different direction [14]: they found quality lowest where the evidence base is contested, we find it lowest where the question itself is unsound. Both implicate reasoning under an unreliable premise rather than recall of settled fact. Neither could be established against a fixed reference: the expert panels used in comparable orthodontic evaluations have themselves agreed only moderately [15], which is the difficulty a construction-based ground truth is intended to remove. The domain analysis sharpens this: what mattered was not how often a domain contained a fabrication but what kind of thing was fabricated. Fabricated protocols and concepts produced clinically consequential elaboration; unverifiable citations and attributions, equally frequent in domain D, produced none.

### 4.4. Citation Criteria and What They Measure

Our comparison of citation criteria quantifies a methodological choice that is rarely made explicit. Counting citations that were never successfully checked as though they had been found absent inflated prevalence from 74.2% to 89.8%. These were not ambiguous or partial matches: the API request failed, so the citation was never assessed. That is a property of the retrieval pipeline, not of the model, and studies that do not separate the two attribute pipeline limitations to models.

The converse caution deserves the same scrutiny, and we tested it rather than assuming it. A non-retrievable citation is not a demonstrated fabrication as a matter of logic, since PubMed and CrossRef do not index every legitimate source. In this dataset, however, the two coincided completely where we could check: all 143 DOIs from responses in which nothing could be verified were absent from both CrossRef and the DOI resolver. That does not licence treating non-retrieval as fabrication in general, and it says nothing about citations given without a DOI, but within this benchmark the automated verdict and a direct manual check agreed in every case. The separation of the two criteria is therefore preserved, and now carries evidence behind it. The strict definition counts only citations for which retrieval completed and returned nothing. In the subset we could check directly, those citations were absent from the scholarly record rather than merely missing from the databases we queried. Four things are worth keeping apart throughout. Failure to verify a citation is a property of the databases queried. An unsupported factual claim is a property of the response. The model’s expression of uncertainty is a property of how the response is phrased. Potential clinical consequence is our author-defined judgement about what a clinician acting on the content might do. Only the second is measured directly; the first depends on retrieval coverage, the third on a lexical rule, and the fourth on the tier mapping.

The adjudication of numeric claims makes the same point from a second direction. Two-thirds of responses flagged as pseudo-quantitative stated clinically defensible values that lacked a traceable source, and only one in six stated a value that was actually wrong. An automated flag treating “unsourced” as “wrong” over-identifies error roughly five to one. Both authors reached the same judgement on every response in this respect (κ = 1.00 for the incorrect-versus-not distinction), so the correction does not reflect one rater’s standard.

We initially examined epistemic hedging as a candidate bedside marker, since it is observable without verification infrastructure. We do not report it as one. The apparent association is largely an artefact of the coding rule, and the one Tier 1 component free of that rule shows no relationship. Whether hedging carries information about clinical risk remains open and would require a taxonomy in which hedging is not part of any severity-defining criterion.

### 4.5. Limits of a Severity Framing

A tier structure that separates clinical from epistemic consequence does not licence reliance on the epistemic tier. Three cautions follow. First, an error without immediate treatment implications may still propagate: a fabricated citation supporting an otherwise sound recommendation removes the reader’s means of checking it, and a diagnostic inaccuracy with no direct consequence may alter a subsequent decision it feeds into. Our design measures the response, not the downstream decision path, and cannot bound that propagation. Second, the frequency finding is not reassuring, and we do not present it as such. The most conservative model still produced at least one unsupported element in 73% of responses, and that is the result a clinician should carry away. The severity distribution tells them where to look first; it does not tell them that most output can be trusted. Third, clinical credibility is not divisible in the way the tiers are. A clinician who repeatedly made unsupported factual claims would lose standing regardless of whether any single claim changed a treatment decision, and the same standard reasonably applies to a system consulted in that role. The argument for severity stratification is that it makes evaluation more informative, not that it makes the underlying error rate acceptable.

A further limit concerns the systems evaluated. All five are general-purpose models with no dental specialisation, no retrieval grounding and no curated clinical corpus. Domain-adapted assistants marketed for dentistry, and retrieval-augmented configurations answering only from a fixed source set, may behave differently. Unverifiable citations would plausibly be fewer, since retrieval constrains what can be cited. Fabricated protocols need not be: a retrieval layer does not by itself supply the judgement needed to reject a false premise. Our benchmark is directly reusable on such systems and the comparison would be informative; we did not make it, and nothing here should be read as characterising specialised tools.

### 4.6. Limitations

The taxonomy constrains where Tier 1 can occur. H3, which defines most of Tier 1, fires only on items carrying a fabricated element and only when hedging is absent. Consequently, the contrast between fabricated-element and other items is partly determined by the coding rules, and the hedging contrast almost entirely so. We have reported both with that constraint stated rather than presenting either as an unconditioned effect. The comparisons least affected are the between-model comparison, in which every model faced identical items, and the within-fabricated-element domain comparison, in which the constraint applies equally to all groups.

The most important limitation is that severity was assigned at the level of taxonomy category rather than by independent clinical review of each response. No clinician outside the author team rated the clinical significance of any individual response. The mapping was fixed before tier-level analysis, and its influence is bounded in Table 5, but the central quantity of this paper—the proportion of responses classed as clinically consequential—rests on an author-defined rule rather than on external clinical judgement, and should be read accordingly. A response-level severity rating by clinicians blind to model identity is the necessary next step, and until it is done the absolute figure should be treated as provisional even though the comparative findings are less sensitive to it.

The stratification was specified after the primary analysis. It reanalyses the same dataset using the taxonomy set out in the deposited protocol, but the tier structure itself was not part of that protocol and should be treated as exploratory. As noted in Section 2.1, the protocol deposit followed the first day of data collection, and the item-level benchmark was deposited only on completion, so the study is pre-specified rather than prospectively registered.

H2 was assigned to the operational tier on the reasoning that a fabricated appliance costs time rather than altering a treatment decision. A clinician who substitutes a non-existent device for an appropriate one has made a clinical error, so the boundary is defensible but not uniquely so; Tier 1 and Tier 2 counts are reported separately for readers applying a different boundary.

Adjudication of numeric claims was performed by the two authors rather than by clinicians external to the study. Raters were blinded to model identity and query identifier, and agreement on the tier-defining distinction was complete, but external adjudication would be stronger.

Two design constraints deserve more weight than their position here might suggest. Each query was issued once per model; providers do not guarantee determinism even at temperature = 0, so run-to-run variability was not estimated, and every rate reported carries an unquantified component of sampling variation from the models themselves. Gemini 2.5 Flash was queried through the standard generateContent interface with only temperature and max_output_tokens set, so no extended-reasoning budget was requested and the model answered under whatever default the provider applied at the time. Its results characterise that call configuration rather than the model in general, and its position relative to the others is not a ranking of the product as ordinarily used. A run with extended reasoning explicitly enabled would be required before the comparison could be read as one between model families. Repeated administration and a default-configuration arm would both be required before either limitation could be bounded. Both sit inside a narrower boundary that applies to everything reported here: the findings describe specific model snapshots queried through specific API configurations over an eight-day window in mid-2026. Provider-side updates, changes to system-level prompts or safety layers, the addition of retrieval features, regional differences in deployment, and repeated sampling could each move these rates materially. Statements about a named model family, as opposed to the particular build and configuration we queried, are not supported by this design, and we avoid making them.

## 5. Conclusions

Hallucination prevalence and clinically consequential error are not interchangeable. In this dataset, they differed by a factor of seven, and the two measures did not rank the models the same way: the 27-point separation on prevalence contracted to 6 points at the clinical tier, where no pair of models could be distinguished once multiplicity was controlled. Item-level clustering was strong for clinically consequential output, meaning that responses to the same item resembled one another closely; this reflects heterogeneity between items rather than a demonstration that the model is irrelevant. Within this benchmark, such output arose almost entirely in response to items containing a fabricated premise—a contrast partly determined by the coding rules, as set out above—and its severity depended on what kind of entity had been fabricated. Reporting severity-stratified rates alongside aggregate prevalence, and separating confirmed non-retrievable citations from those that could not be checked, would make LLM evaluations in clinical domains more directly usable by the clinicians who must act on them.

## Figures and Tables

**Table 3:** Hallucination rates by model with Wilson 95% confidence intervals for each severity tier. n = 100 responses per model. Tiers are not mutually exclusive, so a response may contribute to more than one column.

Model	Any (%)	Tier 1 (%)	Tier 1 95% CI	Tier 2 (%)	Tier 2 95% CI	Tier 3 (%)	Tier 3 95% CI
Claude Opus 4.5	73	10	5.5–17.4	17	10.9–25.5	66	56.3–74.5
Gemini 2.5 Flash	85	11	6.3–18.6	15	9.3–23.3	69	59.4–77.2
DeepSeek-V3	95	15	9.3–23.3	23	15.8–32.2	91	83.8–95.2
GPT-4o	96	13	7.8–21.0	20	13.3–28.9	94	87.5–97.2
Llama 3.3 70B	100	16	10.1–24.4	23	15.8–32.2	100	96.3–100.0

**Table 4:** Tier 1 rates among responses to items containing a fabricated element, by query domain. Domain A (clinical protocols) contained no fabricated-element items and is therefore not represented; its three Tier 1 responses were adjudicated numeric errors. Percentages are response-level; the formal comparison in Section 3.4 is item-level.

Query Domain	Trap Items	Responses	Tier 1 n	Tier 1%
B—Materials and appliances	9	45	3	7
C—Cephalometric and biomechanical	8	40	28	70
D—Cases and landmark studies	8	40	0	0
E—Adversarial items	20	100	29	29

**Table 5:** Clinical tier prevalence under six alternative severity definitions. Ratio compares undifferentiated prevalence (89.8%) with the clinical tier under each definition. Model *p* is Cochran’s Q across the five models, which accounts for the matched item structure.

Definition of the Clinical Tier	*n*	%	95% CI	Ratio to 89.8%	Model Q (*p*)
H3 alone (fabricated protocols)	57	11.4	8.9–14.5	7.9×	9.43 (0.051)
H3 + H5	58	11.6	9.1–14.7	7.7×	5.75 (0.219)
H3 + H5 + adjudicated numeric errors (reported)	65	13.0	10.3–16.2	6.9×	10.00 (0.040)
+ H2 (fabricated devices)	124	24.8	21.2–28.8	3.6×	20.18 (<0.001)
+ all H6 (any unsourced numeric claim)	165	33.0	29.0–37.2	2.7×	29.56 (<0.001)
Maximal (all categories except H1 alone)	218	43.6	39.3–48.0	2.1×	33.75 (<0.001)

## Data Availability

The deposited protocol, query benchmark, raw responses, coded dataset, and statistical output are openly available at the Open Science Framework (DOI 10.17605/OSF.IO/DK2PM). Analysis code is available at https://github.com/drmustafaozcan/llm-ortho-hallucination (accessed on 27 September 2026) under an MIT licence.

## Supplementary Materials

The following supporting information can be downloaded at: https://www.mdpi.com/article/10.3390/diagnostics16193180/s1, Table S1: External content-validity panel and item-level ratings. Table S2: Fabricated entity types by query domain. Table S3: Operational coding criteria. Table S4: full statistical output. Table S5: Item-level adjudication of unsupported numeric claims. Table S6: Citation verification outcomes by model. File S1: Verbatim response excerpts illustrating hallucination categories and hedging contrasts. File S2: Completed CHART and STROBE reporting checklists.
